# Differential modulation of collybistin conformational dynamics by the closely related GTPases Cdc42 and TC10

**DOI:** 10.3389/fnsyn.2022.959875

**Published:** 2022-08-04

**Authors:** Nasir Imam, Susobhan Choudhury, Katrin G. Heinze, Hermann Schindelin

**Affiliations:** ^1^Institute of Structural Biology, Rudolf Virchow Center for Integrative and Translational Bioimaging, University of Würzburg, Würzburg, Germany; ^2^Molecular Microscopy, Rudolf Virchow Center for Integrative and Translational Bioimaging, University of Würzburg, Würzburg, Germany

**Keywords:** autoinhibition, fluorescence resonance energy transfer (FRET), gephyrin, guanine nucleotide exchange factor (GEF), inhibitory postsynapse, Rho GTPase

## Abstract

Interneuronal synaptic transmission relies on the proper spatial organization of presynaptic neurotransmitter release and its reception on the postsynaptic side by cognate neurotransmitter receptors. Neurotransmitter receptors are incorporated into and arranged within the plasma membrane with the assistance of scaffolding and adaptor proteins. At inhibitory GABAergic postsynapses, collybistin, a neuronal adaptor protein, recruits the scaffolding protein gephyrin and interacts with various neuronal factors including cell adhesion proteins of the neuroligin family, the GABA_*A*_ receptor α2-subunit and the closely related small GTPases Cdc42 and TC10 (RhoQ). Most collybistin splice variants harbor an N-terminal SH3 domain and exist in an autoinhibited/closed state. Cdc42 and TC10, despite sharing 67.4% amino acid sequence identity, interact differently with collybistin. Here, we delineate the molecular basis of the collybistin conformational activation induced by TC10 with the aid of recently developed collybistin FRET sensors. Time-resolved fluorescence-based FRET measurements reveal that TC10 binds to closed/inactive collybistin leading to relief of its autoinhibition, contrary to Cdc42, which only interacts with collybistin when forced into an open state by the introduction of mutations destabilizing the closed state of collybistin. Taken together, our data describe a TC10-driven signaling mechanism in which collybistin switches from its autoinhibited closed state to an open/active state.

## Introduction

In the central nervous system, synaptic neurotransmission is mediated by ligand-gated ion channels which are assembled at postsynaptic specializations. The postsynaptic localization of ion channel receptors is vital for efficient synaptic neurotransmission and the precise regulation of distinct neuronal functions (Andersen et al., 1963; Buhl et al., 1994; Nusser et al., 1997). Inhibitory neurotransmission is mediated by glycine and gamma amino-butyric acid (GABA) and cognate receptors for these neurotransmitters are recruited and stabilized by the scaffolding protein gephyrin (Betz, 1998; Fritschy et al., 2008; Tyagarajan and Fritschy, 2014). Gephyrin has been postulated to form extended structures beneath the plasma membrane, where its interaction with the receptors stabilizes the receptors and inhibits their lateral movement (Kneussel and Betz, 2000; Moss and Smart, 2001). Gephyrin recruitment from intracellular deposits to the plasma membrane mainly relies on the adaptor protein collybistin (CB; alternatively known as ARHGEF9) (Kins et al., 2000; Papadopoulos and Soykan, 2011; Soykan et al., 2014).

Collybistin belongs to the diffuse B-cell lymphoma (Dbl) family of guanine nucleotide exchange factors (GEFs) (Zheng, 2001). The murine CB gene is expressed in three splice variants (CB1-CB3) which differ in the presence or absence of a regulatory src homology 3 (SH3) domain and their C-terminal residues (Harvey, 2004). In addition to the SH3 domain, all CB splice variants contain tandem Dbl homology (DH) and pleckstrin homology (PH) domains, which are responsible for its role as a GEF and plasma membrane tethering, respectively (Xiang et al., 2006; Reddy-Alla et al., 2010; Papadopoulos and Soykan, 2011; Ludolphs et al., 2016). GEFs play essential roles in the reactivation of RAS homologue (Rho)-like GTPases (Xiang et al., 2006; Sinha and Yang, 2008; Papadopoulos and Soykan, 2011), which ensures that these GTPases play important roles in regulating cytoskeletal rearrangements, cell motility, cell polarity, axon guidance, vesicle trafficking, and the cell cycle (Heasman and Ridley, 2008; Hodge and Ridley, 2016).

Previous studies demonstrated that the most widely expressed, SH3-domain containing CB isoform-2 splice variant (CB2-SH3^+^) preferentially adopts a closed conformation, in which the N-terminally located SH3 domain interacts intra-molecularly with the tandem DH-PH domains (Soykan et al., 2014). Cellular data suggested that all SH3 domain-encoding CB variants remain untargeted and colocalize with intracellular gephyrin deposits and hence require additional factors including NL2, NL4, or the α2-subunit of the GABA_*A*_ receptor which interact with the SH3 domain, thus inducing an open or active state conformation (Kins et al., 2000; Harvey, 2004; Poulopoulos et al., 2009; Saiepour et al., 2010; Hoon et al., 2011; Soykan et al., 2014; Hines et al., 2018). Here “active” does not refer to the ability of CB to act as a GEF, instead it reflects its ability to contribute to neurotransmitter receptor clustering. The SH3 domain-deficient CB isoform (CB2-SH3^–^), on the contrary, adopts an open conformation, which possesses enhanced postsynaptic gephyrin-clustering and effectively replenishes the GTP-bound state of the small GTPase Cdc42 from its GDP-bound state (Xiang et al., 2006; Reddy-Alla et al., 2010; Chiou et al., 2011; Tyagarajan et al., 2011; Soykan et al., 2014). Additionally, biochemical and cell-based studies suggested that amino-acid replacements weakening the inter-domain association of CB lead to an open/active CB conformation in which the DH domain is exposed (Soykan et al., 2014; Schäfer et al., 2020).

Based on previous biochemical experiments, CB was originally considered to be a Cdc42-specific GEF (Reid et al., 1999). However, contrary to this prevalent assumption, recent studies suggested that CB interacts differently with the closely related small GTPase, TC10 (also referred to as RhoQ), which is 67.4% identical with Cdc42 (Neudauer et al., 1998; Hemsath et al., 2005; Mayer et al., 2013; Kilisch et al., 2020). In contrast to Cdc42, which is ubiquitously expressed in all brain regions, TC10 expression is limited to specific hippocampal regions in the mammalian brain (Tanabe et al., 2000).

The crystal structure of the Cdc42-CB2SH3^–^ complex revealed that CB binds to Cdc42 *via* its catalytic DH domain (Xiang et al., 2006). TC10, however, preferentially interacts with the C-terminally located PH domain of CB (Mayer et al., 2013; Kilisch et al., 2020). Furthermore, *in cellulo* studies suggested that TC10 promotes a CB-dependent gephyrin redistribution, thereby regulating GABAergic postsynaptic strength (Mayer et al., 2013). Although previous studies indicated that TC10 binding to CB interferes with the inter-domain autoinhibitory interactions of CB (Mayer et al., 2013; Kilisch et al., 2020), an understanding of the molecular basis of TC10-mediated CB activation is still lacking. With respect to the closely related Cdc42 it is unclear whether it can activate CB and, if yes, how this is accomplished.

In the present study, we delineate how Cdc42 and TC10 modulate CB conformational dynamics. Through a series of custom engineered CB FRET sensors, we describe the molecular basis of TC10-mediated CB conformational activation. Using time-resolved fluorescence lifetime measurements we demonstrate that TC10 and Cdc42 elicit differential responses in auto-inhibited CB; specifically, TC10, unlike Cdc42, can efficiently induce CB opening. Binding affinity quantification for TC10 shows enhanced affinity for an open state mutant sensor compared to the wild-type CB, whereas Cdc42 binds only to the active state mutant of CB, but with substantially reduced affinity compared to TC10. By analyzing the sequences and structures of the two GTPases we identify molecular determinants for the differential interactions between CB and TC10/Cdc42. Taken together, our data provide a structural framework for TC10-driven CB conformational activation of its auto-inhibited form.

## Materials and methods

### Cloning, expression, purification, and *in vitro* FLAsH labeling

An N-terminal His_6_-tagged wild-type TC10 construct was generated by subcloning the murine cDNA coding for residues 1-205 into the pETM14 vector using restriction free (RF) cloning (Bond and Naus, 2012). TC10KR/GS was subsequently constructed by site-directed mutagenesis. The C-terminal TC10 deletion variant, TC10ΔC, was constructed by deleting the last 20 amino acids by using RF cloning. The full-length Cdc42 construct has been previously described (Xiang et al., 2006) as have the wild-type CB FRET sensor (F1_*D0*_), open state mutant sensors (F1_*smD0*_ and F1_*dmD0*_) and the series of additional CB FRET sensors (Imam et al., 2022). All FRET sensors are derived from the CB2-SH3^+^ variant.

Wild-type TC10 and its C-terminal variants were expressed in the *E. coli* strain BL21 (DE3). Bacterial cell lysates were subjected to affinity chromatography on Protino Ni-IDA resin (Macherey Nagel) equilibrated in lysis buffer (50 mM Tris-HCl pH 8, 250 mM NaCl, and 5 mM β-mercaptoethanol). Immobilized proteins were eluted using lysis buffer containing 300 mM imidazole and were subsequently subjected to size exclusion chromatography on a Superdex 200 column (GE Healthcare). Eluted protein fractions were concentrated to 10–12 mg/ml by ultrafiltration, flash frozen and stored at −80°C for later usage. All CB FRET sensors were purified as described (Imam et al., 2022) as was full-length Cdc42 (Xiang et al., 2006), in this case with minor modifications. All CB FRET sensors were FlAsH labeled as described (Imam et al., 2022).

### Time-resolved setup and data acquisition

A custom-built confocal microscopy setup (IX 71, Olympus, Hamburg, Germany) equipped with a time-correlated single photon counting (TCSPC) system (Hydraharp 400, Picoquant, Berlin, Germany) and with data acquisition by the fluorescence lifetime correlation software SymPhoTime 64 (PicoQuant, Berlin, Germany) was used to measure time resolved data. A 440 nm pulsed laser (LDH-D-C-440, Picoquant) was the excitation laser source, which was coupled through a polarization maintaining single mode fiber (PicoQuant, Berlin, Germany). The laser beam was expanded by a telescope to a diameter of 7 mm to fill the back aperture of the objective (60x water immersion, NA 1.2, Olympus, Hamburg, Germany). For epi-illuminating the sample, a beam splitter (HC458 rpc phase r uf1, AHF) was placed before the objective. In the detection path a 50 μm pinhole (PNH-50, Newport, Darmstadt, Germany) rejected out of focus light and the beam was split *via* a polarizing beam splitter cube (10FC16PB.3, Newport, Darmstadt, Germany) into parallel (VV, detector 1) and perpendicular emissions (VH, detector 2) before being projected on photon counting detectors (2x PMA Hybrid-40, Picoquant, Berlin, Germany). An emission band pass filter (Brightline HC 480/40 AHF, Tübingen, Germany) was placed before the detectors to reject unspecific light. The laser was operated in 20 MHz pulsed mode and the power at the sample was maintained at ∼11 μW, while the temporal resolution was kept at 4 ps. All measurements were conducted on standard glass coverslips (Menzel-Gläser, Braunschweig, Germany; 24 × 40 mm, 1.5). The setup was optimized with a 1 μM solution of Coumarin 343. These measurements also provide the relative detection efficiency in the parallel and perpendicular channels, i.e., the g-factor of the setup. To determine the instrument response function (IRF), a KI-saturated solution of 3 μM fluorescein in double distilled water was measured for 10–15 min. 20 μL of each sample (CB FRET sensor mixed with different ligands) were excited at 440 nm and the donor (CFP) emission between 460 and 500 nm was recorded at room temperature for 5–10 min depending on photon counts. Donor only (D0) and buffer solutions were measured as control samples and for background corrections, respectively. Samples were measured in biological triplicates to calculate average values and standard deviations for each condition.

### Time-resolved fluorescence decay analysis

Time resolved fluorescence intensities were analyzed using the Seidel-Software package.^1^ The VV and VH signals collected in ptu format with the Symphotime 64 software were converted into a single column stack using the Jordi-tool of the software package. All data were exported in 16 ps bins, i.e., 4,096 channels for each detector for a total of 8,192 channels. With a given g-factor, the magic angle fluorescence intensity decays were created and analyzed with the chisurf software (Peulen et al., 2017). The g-factor for the setup was calculated to be 0.98 from the tail fitting of the fluorescence time-resolved decay of Coumarin 343. The decay curves were fitted with a multi-exponential model function using an iterative re-convolution approach (Sanabria et al., 2020; Tsytlonok et al., 2020) as follows


(1) $$F\,\left(t\right)=\sum_{i}a_{i}\,e^{-t/\tau_{i}}$$

where *a_i_* represents the amplitude and τ_*i*_ the lifetime of the corresponding component.

Under ideal conditions the donor-only sample (D0) should show a single component, however, due to local quenching we had to conduct a 3-component fitting as reported earlier (Peulen et al., 2017; Lehmann et al., 2020). The reduced χ^2^-values and the weighted residuals were evaluated to check the goodness of the fit. Time-resolved fluorescence intensities for FlAsH labeled (F1_*DA*_) and F1_*DA*_-ligand complexes were also analyzed by Equation 1 to obtain the species-weighted average fluorescence lifetime.


(2) $$\left\langle \tau \right\rangle =\sum_{i}a_{i}\,\tau_{i},$$

(3) $$\text{where}\,\sum_{i}a_{i}=1.$$

### K_*d*_ determination

To determine the K_*d*_ of F1_*DA*_ interacting with TC10 or Cdc42, we titrated the F1_*DA*_ sensor with different concentrations of the respective ligand and measured the corresponding time-resolved fluorescence intensities. The species-weighted average fluorescence lifetimes were used to calculate the fractional saturation (in %) as follows


(4) $$Fractionalsaturation\left(\%\right),f=\frac{\left\langle \tau_{D\,A,i\,M}\right\rangle -\left\langle \tau_{D\,A,0\,M}\right\rangle }{\left\langle \tau_{D\,A,m\,a\,x}\right\rangle }*100$$

where ⟨τ_*DA*,*iM*_⟩ is the average fluorescence lifetime at concentration *i*, ⟨τ_*DA*,0*M*_⟩ is the mean fluorescence lifetime of the FlAsH labeled CB FRET sensor without addition of ligand and ⟨τ_*DA*,*max*_⟩ is the longest mean fluorescence lifetime of the titration, usually obtained at the highest ligand concentration. The resulting data points were plotted against the ligand concentration and fitted as follows (Origin9, OriginLab):


f⁢(x)

$$\;=b+{\left(a-b\right)}^{*}\,\frac{[\left(C_{p}*K_{d}*x\right)\pm \sqrt{{\left(C_{p}*K_{d}*x\right)}^{2}-4^{*}\,C_{p}*x}}{2*C_{p}}$$

Where *x* is the concentration, *b* the offset, *a* the final intensity, *c_p_* the protein concentration, and *K_d_* the dissociation constant.

### Average fluorescence resonance energy transfer efficiency calculation

The average FRET efficiency (E_*FRET*_) is calculated from the average fluorescence lifetimes using the following equation:


(6) $$E_{F\,R\,E\,T}=1-\left\langle \tau_{D\,A}\right\rangle /\left\langle \tau_{D\,0}\right\rangle$$

where ⟨τ_*D*0_⟩ and ⟨τ_*DA*_⟩ are the species-weighted average fluorescence lifetimes in the absence (D0) and presence (DA) of FlAsH as calculated based on Equation 2.

### Förster distance calculation

To determine the inter-fluorophore distance distribution from the fluorescence intensity decays the Förster distance R_0_ needs to be calculated accurately. R_0_ [Å] was calculated from the following equation


(7) $$R_{0}=0.211*{\left[k^{2}\,\eta^{-4}\,\Phi_{D}\,J\,\left(\lambda \right)\right]}^{\frac{1}{6}}$$

where *κ^2^* is a factor describing the relative orientation in space of the transition dipoles of the donor and the acceptor. The magnitude of *κ^2^* is assumed to be 0.66 for a random orientation of donor and acceptor. The refractive index (η) of the aqueous buffer is assumed to be 1.33. The quantum yield (Φ_*D*_) of the donor CFP is 0.4. *J(λ)* is the overlap integral of emission of donor (CFP), and absorption of acceptor (FlAsH) and is calculated by


(8) $$J\,\left(\lambda \right)=\frac{\int_{0}^{\infty }I_{D}\,\left(\lambda \right)\,\varepsilon \,\left(\lambda \right)\,\lambda^{4}\,d\lambda }{\int_{0}^{\infty }I_{D}\,\left(\lambda \right)\,d\lambda }$$

Here, *I_*D*_(λ)* is the fluorescence emission of the donor in the wavelength region λ and *ε(λ)* the extinction coefficient in units of [M^–1^cm^–1^] of the acceptor FlAsH (41000 M^–1^ cm^–1^ at 508 nm).

### Fluorescence resonance energy transfer distance distribution analysis

To accurately determine the inter-fluorophore distance distribution from the fluorescence intensity decays of the FlAsH labeled (F1_*DA*_) and F1_*DA*_-ligand complexes we followed a method described earlier (Sanabria et al., 2020; Tsytlonok et al., 2020). The time-resolved fluorescence intensities of the FRET-sample and the donor-only (reference) sample can be represented as:


$$F_{F\,R\,E\,T}\left(t\right)=N_{0}[\left(1-x_{N\,o\,F\,R\,E\,T}\right)F_{D\,A}\left(t\right)$$

(9) $$+x_{N\,o\,F\,R\,E\,T}F_{D\,0}\left(t\right)]⊗IRF+sc*IRF+c$$

(10) $$F_{R\,e\,f}\,\left(t\right)=N_{0}\,F_{D\,0}\,\left(t\right)⊗I\,R\,F+s\,c*I\,R\,F+c$$

where *N*_0_ is the total photon number, *c* the constant offset of the fluorescence intensity, *sc* the scattered light from the sample, and *x*_*NoFRET*_ the no-FRET contribution from the unquenched donor. As stated earlier, we obtained multi-exponential fitting for the donor-only sample due to local quenching, however, the local quenching of the donor is not affected by FRET (Lehmann et al., 2020). Thus, the FRET-rate (*k*_*FRET*_) depends on the relative orientation of the fluorophores and donor-acceptor-distance and the FRET samples can be fitted globally with the donor-only reference sample. In the presence of FRET, the donor fluorescence decay can be expressed with a Gaussian distance distribution (ρ) of the donor-acceptor pair as


$$F_{D\,A}\,\left(t\right)=F_{D\,0}\,\left(t\right)*\int \rho_{G\,a\,u\,s\,s}\,\left(\sigma \,\left\langle R\,\left(i\right)\right\rangle \right)$$

(11) $$\,*\text{exp}\,\left(-k_{F\,R\,E\,T}\,\left(R\,\left(i\right)\right)*t\right)\,d\,R$$

where ⟨*R*(*i*)⟩ is the mean distance between donor and acceptor and σ the width of the inter-fluorophore distance distribution*R*(*_i_*). The calculated Förster radius for CFP and the FlAsH pair was 39 Å and σ was kept fixed to a physically meaningful value of 5 Å (Peulen et al., 2017).

### Uncertainty estimation of distance distribution

There are three sources of the experimental uncertainty in the TCSPC-based inter-fluorophore distance distribution analysis: (i) Orientation factor (κ^2^) uncertainty, δR_*DA*κ2)_, (ii) the uncertainty in the D_*only*_ reference δR_*DA*_, reference (based on sample preparation, etc.), and (iii) the statistical distance distribution fitting uncertainty, δR_*DA,fit*_ (Peulen et al., 2017). To estimate the uncertainty δR_*DA,fit*_ we sampled the *χr*^2^-surface of the fit over the range −20% to +20% in 50 steps of the respective distance using the “Parameter Scan” option in ChiSurf (Peulen et al., 2017). The resulting *χr*^2^-surface (Lakowicz, 2013) was plotted against the scanned distance and the limits were determined using a 3σ-criterion based on an F-test (1700 TCSPC channels, 9 parameters) to a relative *χr*,_*rel*_^2^ = *χr*,_*i*_^2^/*χr*,_*min*_^2^ of 1.012. To evaluate δR_*DA,reference*_, we had extended the limits for R_*min*_ and R_*max*_ in such a way that the overall R_*min*_ and R_*max*_ for the experimental triplicates were used. The uncertainty of the orientation factor (κ^2^), δR_*DA*κ2)_, which is usually the largest source of uncertainty, was not considered.

### Model free distance distribution analysis

For the model-free distance distribution analysis, we calculated the FRET-induced donor decay as described earlier (Peulen et al., 2017). Briefly, as a first step, the fluorescence decay of the FRET sample *I*_*DA*_(*t*) is divided by the (fit) decay of the donor-only sample *I*_*D0*_(*t*). Next, the D_*Only*_ fraction, *x*_*NoFRET*_, i.e., the offset of the decay, is subtracted, and finally, this ratio is multiplied with the time axis *t* to yield the FRET-induced donor decay ε(*t*):


(12) $$\varepsilon \,\left(t\right)=\left(\frac{I_{D\,A}\,\left(t\right)}{I_{D\,0}\,\left(t\right)}-x_{N\,o\,F\,R\,E\,T}\right)*t$$

For an intuitive display, we converted the x-axis from time *t* to critical distance *R*_*DA,c*_ by the following relation:


(13) $$R_{D\,A,c}=R_{0}*{\left(\frac{t}{\tau_{D}}\right)}^{1/6}$$

Here *R*_0_ is the Förster radius of the respective FRET dye pair (39 Å in this case) and *τ_*D*_* the reference fluorescence lifetime of the donor fluorophore (here, 3.1 ns). Plotting ε(*t*) against *R*_*DA,c*_ results in a peak distribution, which reflects the probability density function of the underlying distance distribution of the original decay *I*_*DA*_(*t*).

## Results

### Comparative analysis of TC10 and Cdc42 structures

**FIGURE 1:**
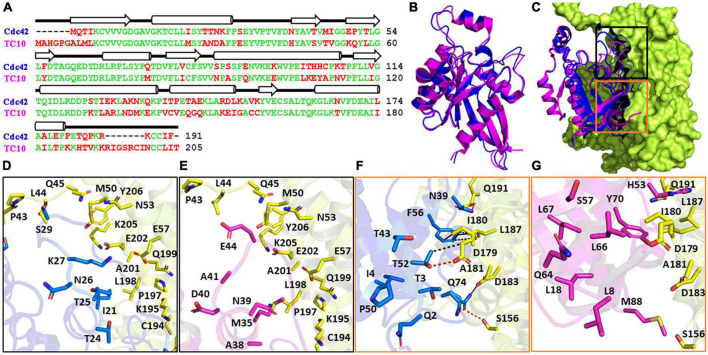
Cdc42 and TC10 comparison. **(A)** Sequence alignment calculated with Clustal (Sievers et al., 2011) of murine TC10 and Cdc42. Conserved amino acid residues are displayed in green, diverging residues in red. On the top, β-strands (arrows) and α-helices (cylinders) are indicated. **(B)** Superimposition of Cdc42 (PDB entry 1an0; blue) and TC10 (PDB entry 2atx; magenta), highlighting their conserved three-dimensional structures. **(C)** Crystal structure of the Cdc42-CB2SH3^–^ complex (PDB entry 2dfk) superimposed with TC10 (PDB entry 2atx). TC10 (magenta) and Cdc42 (blue) are shown in cartoon, whereas CB2SH3^–^ (lemon) is depicted in surface representation. Black and orange boxes represent the top and bottom portion of the Cdc42-CB interface. **(D,E)** Zoomed image of the top interface region of the CB-Cdc42 complex **(D)** and the hypothetical CB-TC10 interface region following superimposition of TC10 onto Cdc42 **(E)**. Residues of Cdc42, which are part of the interface but are not conserved in TC10, are represented with their side chains in blue **(D)** and the corresponding residues of TC10 in magenta **(E)**. **(F,G)** Enlarged image of the bottom section of Cdc42-CB interface **(F)** and that of the hypothetical TC10-CB complex **(G)**. Residues are highlighted as described for panels **(D,E)**. Hydrogen bonds and hydrophobic interactions in the Cdc42-CB complex **(F)** are represented by red and black dotted lines, respectively.

The CB interacting GTPases TC10 and Cdc42 are closely related (Figure 1A) which is reflected in a high amino-acid sequence identity of 67.4% (Neudauer et al., 1998). Despite the high conservation, the N and C-termini of both GTPases contain small stretches of non-conserved residues. Furthermore, additional short patches of non-identical residues can be observed in the core regions of the GTPases (Figure 1A). As expected, the superimposition of both GTPases (Figure 1B) revealed a high degree of structural similarity as reflected in a root mean square (RMS) deviation of 0.52Å for the Cα-atoms. To understand why TC10 does not interact in the same way with CB as Cdc42, we superimposed TC10 onto the crystal structure of the Cdc42-CBSH3^–^ complex (Xiang et al., 2006; Figure 1C) and analyzed the distribution of non-conserved residues in the interface of the complex (Figures 1C–G).

The interface can be divided into two areas, designated as top and bottom, where non-conserved residues are observed. Figures 1D,E represent the top section of the interface for Cdc42 (based on the crystal structure) and TC10 (based on the superimposition), respectively. In this region six non-identical residues between Cdc42 and TC10 are observed. The bottom part of the Cdc42-CBSH3^–^ interface (Figure 1F) and the hypothetical TC10-CBSH3^–^ interface (Figure 1G) features nine non-identical residues. An analysis of the protein-protein interface with PDBePISA (Krissinel and Henrick, 2007) revealed that the residues present in the top interface in the Cdc42-CBSH3^–^ complex do not form any hydrogen bonds (Figure 1D). The side chains of the non-conserved residues were also predicted not to be involved in any van der Waals’ interactions. In contrast, in the bottom interface, the non-conserved residues N39, T52, and Q74 of Cdc42 (Figure 1F) engage in hydrogen bonds with Q191, D179, and S156 of CB, respectively. The corresponding residues in TC10, H53, L66, and M88 (Figure 1G), either lack the potential to form hydrogen bonds (L66 and M88) or, due to size differences (H53), can no longer form hydrogen bonds. Moreover, the hydrophobic interactions between F56 in Cdc42 and I180 as well as L187 in the DH domain of CB are weakened by the substitution of Y70 in TC10 for F56 in Cdc42. Please note that eight non-native residues (SPGAGRSS) are present at the N-terminus of TC10 (PDB entry 2atx) (Hemsath et al., 2005), which are partially responsible for the offset in residue numbers (Figure 1A). This analysis indicates that the aforementioned substitutions mediate the differential binding preferences of Cdc42 and TC10 to the DH-PH tandem of CB (Xiang et al., 2006; Mayer et al., 2013) and explains why TC10 cannot be bound in a manner analogous to Cdc42. Since the interaction between the PH domain of CB and TC10 has not yet been structurally characterized, it is unclear which residues in either protein are involved and why Cdc42 cannot engage in the same interaction with the PH domain of CB.

### TC10 mediates conformational activation of auto-inhibited collybistin

**FIGURE 2:**
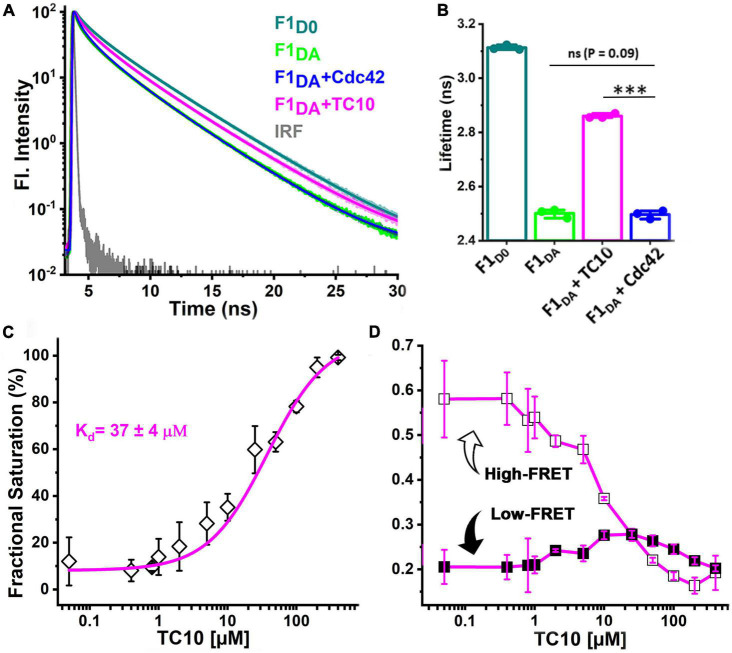
TC10 and Cdc42 interaction with a CB FRET sensor. **(A)** Time-resolved CFP fluorescence intensities for the CB FRET sensor F1_*D0*_ (teal), its FlAsH-labeled counterpart F1_*DA*_ alone (green) and in the presence of a 100-fold molar excess (100 μM) of Cdc42 (blue) and TC10 (magenta). The instrument response function (IRF) is shown in gray. F1_*D0*_ and F1_*DA*_ were excited (λ_*ex*_) at 440 nm. Emission (λ_*em*_) data were collected between 460 and 500 nm and fitted with Equation 1 to obtain the average fluorescence lifetime ⟨τ⟩ for the respective samples. For easier comparison data were scaled to a maximum of 10^2^. **(B)** Bar plot showing species-weighted ⟨τ⟩ of CFP in F1_*D0*_ (teal), F1_*DA*_, alone (green) and in the presence of a 100-fold molar excess of TC10 (magenta) and Cdc42 (blue). Data from three individual biological replicates (*n* = 3) are presented as mean values ± SD. ^***^P < 0.001; ns, statistically not significant. **(C)** F1_*DA*_ binding affinity plot of TC10. Binding affinity was determined by first converting ⟨τ⟩ into the fractional saturation using Equation 4 and the data were further fitted with Equation 5. The F1_*DA*_ binding affinity constant (K_*d*_) for TC10 was measured as 37 ± 4 μM. **(D)** Plot showing the FRET species (high-FRET state and low-FRET state) composition plotted against increasing concentrations of TC10, obtained after analyzing the time-resolved fluorescence intensities with the Gaussian distribution model (Equations 9–11). With increasing concentrations of TC10, the high-FRET state (R_1_, open square) gradually decreases, while the low-FRET (R_2_, filled square) state increases.

In order to examine the role of the two GTPases in CB activation, we employed previously described fluorescence lifetime-based CB FRET sensors (Imam et al., 2022) derived from the CB2-SH3^+^ splice variant (Supplementary Figure 1A). We recombinantly purified TC10 and Cdc42 (Supplementary Figures 1B,C) and incubated both proteins in a 100-fold molar excess (100 μM) with the CB wild-type FRET sensors (Imam et al., 2022). For interaction studies, we individually measured the average fluorescence lifetime (⟨τ⟩) of CFP (Figure 2A) of the CB FRET sensor (F1_*D0*_) and its FlAsH-labeled counterpart (F1_*DA*_), in the absence and presence of either TC10 or Cdc42. Time-resolved fluorescence intensities of CFP in the presence of FlAsH (F1_*DA*_) showed a significant ⟨τ⟩ reduction, from 3.1 ± 0.03 ns (mean ± standard deviation; SD) to 2.52 ± 0.02 ns (Table 1). When incubated with F1_*D0*_ neither TC10 nor Cdc42 induced any change in ⟨τ⟩ of CFP (Table 1), thus indicating that both GTPases do not alter the fluorescence characteristics of the C-terminally attached CFP in the F1_*D0*_. Interestingly, upon incubation of F1_*DA*_ with TC10 a substantial increase (2.87 ± 0.01 ns) in the ⟨τ⟩ of F1_*DA*_ was observed (Figures 2A,B and Table 1). The TC10-induced ⟨τ⟩ increase in the F1_*DA*_ can be attributed to an inter-dye distance increase between the donor fluorophore, CFP, and the acceptor fluorophore, FlAsH. In contrast, Cdc42 did not show a significant ⟨τ⟩ change in F1_*DA*_ compared to free F1_*DA*_ (Figures 2A,B and Table 1). The unaltered F1_*DA*_ ⟨τ⟩ in the presence of Cdc42 (2.53 ± 0.03 ns) suggests that the SH3-containing CB2 variant, which is known to be in an autoinhibited state (Soykan et al., 2014), at best only weakly interacts with Cdc42.

**TABLE 1:** Table representing the species-weighted average fluorescence lifetime (⟨τ⟩) and inter-fluorophore distances (R_*i*_) along with their relative species fractions (x_*i*_) obtained from time-resolved FRET analysis for the CB-FRET sensors (F1_*D0*_ and F1_*DA*_) alone and after incubation with a 100-fold molar excess (100 μM) of Cdc42, TC10, and its C-terminal variants.

Sample	⟨τ⟩ (±SD), [ns]	R_1_ (±SD) [Å]	X_1_ (±SD)	R_2_ (±SD) [Å]	X_2_ (±SD)	x_*NoFRET*_ (±SD)
F1_*D0*_	3.1 (±0.03)	–	–	–	–	–
F1_*DA*_	2.52 (±0.02)	25.5 (±1.5)	0.45 (±0.02)	45.5 (±0.9)	0.21 (±0.02)	0.32 (±0.03)
F1_*D0*_ + Cdc42	3.12 (±0.02)	–	–	–	–	–
F1_*D0*_ + TC10	3.1 (±0.01)	–	–	–	–	–
F1_*DA*_ + Cdc42	2.53 (±0.03)	26.2 (±1.2)	0.48 (±0.02)	42.5 (±1.9)	0.21 (±0.04)	0.29 (±0.11)
F1_*DA*_ + TC10	2.87 (±0.01)	26.8 (±2.6)	0.20 (±0.01)	47.5 (±4.3)	0.23 (±0.09)	0.68 (±0.18)
F1_*DA*_ + TC10KR/GS	2.89 (±0.03)	25.3 (±1.9)	0.16 (±0.06)	45.1 (±4.0)	0.17 (±0.06)	0.67 (±0.17)
F1_*DA*_ + TC10ΔC	2.83 (±0.02)	27.1 (±1.1)	0.18 (±0.1)	48.1 (±0.6)	0.24 (±0.1)	0.56 (±0.13)

Species fractions are normalized so that x_1_ + x_2_ + x*_NoFRET_* = 1. Data from three individual biological replicates (*n* = 3) are presented as mean values ± SD.

### TC10 stabilizes an open conformation of collybistin

To further characterize the CB-TC10 interaction we carried out titration experiments to determine the binding affinity between TC10 and CB by incubating increasing concentrations (0.05–400 μM) of TC10 with F1_*DA*_, while keeping the F1_*DA*_ concentration constant. With increasing TC10 concentrations, F1_*DA*_ showed a consequent increase in ⟨τ⟩, finally reaching saturation at higher molar concentrations of TC10 (Supplementary Figure 2A). We plotted the fractional saturation determined from the corresponding ⟨τ⟩ change (Equation 4) against the TC10 concentration and determined a dissociation constant (K_*d*_) of the F1_*DA*_-TC10 complex of 37 ± 4 μM (Equation 5 and Figure 2C), suggesting a moderately tight interaction between CB and TC10. Since for Cdc42 no change in ⟨τ⟩ was observed, even at significantly higher concentrations (Figures 2A,B and Table 1), the binding strength could not be measured.

To further investigate the role of TC10 in CB activation we analyzed the time-resolved fluorescence intensities of F1_*DA*_ and F1_*DA*_-TC10 complexes at varying concentrations by Gaussian distance distribution models (Equations 9–11) as described before (Imam et al., 2022). Consistent with our previous study, F1_*DA*_ molecules adopted two distinct conformational states, a high-FRET state exhibiting a compact conformation and a low-FRET state reflecting an open conformation (Figure 2D; Supplementary Figure 3A). The inter-fluorophore distances (Table 1) in the high (R_1_) and low FRET states (R_2_) were calculated as 26.8 ± 2.6Å and 47.5 ± 4.3Å, respectively. Increasing TC10 concentrations resulted in a significant shift in the equilibrium from the high to the low FRET state (Figure 2D; Supplementary Figure 3A). Interestingly, higher TC10 concentrations also led to a stronger population of a NoFRET (x_*NoFRET*_) state (Supplementary Figure 4A), possibly indicating another state beyond the measurable FRET distance limit (>49Å) for the FRET pair used in this study. The fluorescence lifetime-based FRET study along with distance distribution analysis of F1_*DA*_ provided concrete evidence of a TC10-mediated CB opening and its transition from the closed to an open state.

### TC10 C-terminal variants efficiently recognize collybistin

Small GTPases possess variable C-terminal regions which contain diverse types of subcellular localization signals and harbor sites for various post-translational modifications (Michaelson et al., 2001; Murphy et al., 2001; Watson Robert et al., 2003; Roberts et al., 2008; Lionel et al., 2013). Specifically, most Rho GTPases at their C-termini possess a stretch of basic residues which is believed to mediate their positioning at the appropriate cellular membrane sites to ensure proper signal transduction (Hodge and Ridley, 2016). In line with this observation, the C-terminal tail of TC10 also contains the previously described cluster of positively charged residues, which serves as a binding site for various phosphoinositides (Kilisch et al., 2020).

**FIGURE 3:**
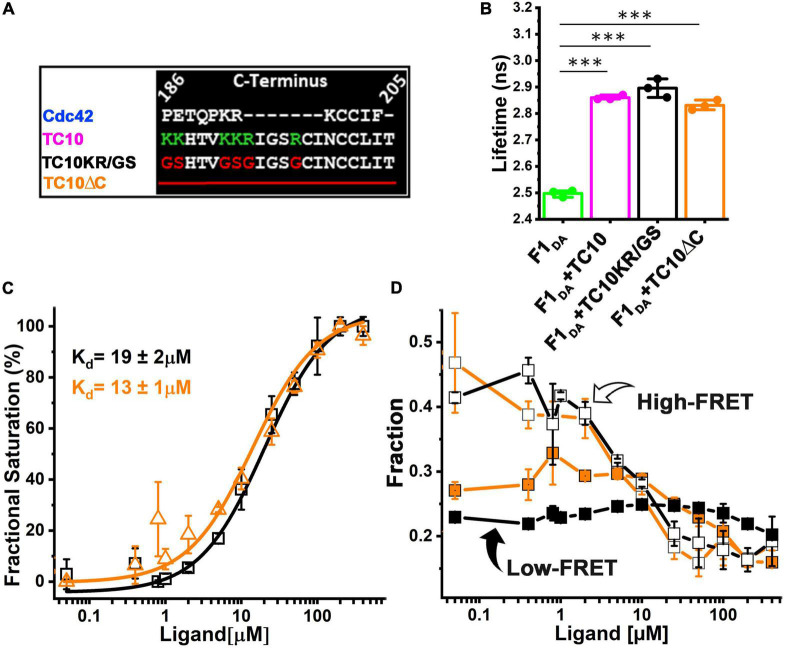
CB binding affinity for TC10 and variants impaired in phosphoinositide-binding. **(A)** Alignment of the C-terminal residues of Cdc42 and TC10 (wild-type and variants). In the TC10KR/GS variant the basic residues K and R (shown in green) were replaced with G and S (red). In case of TC10ΔC, the C-terminal amino acid stretch was completely removed. **(B)** Bar plots depicting the species-weighted average CFP fluorescence lifetime of F1_*DA*_ alone (green) and in the presence of a 100-fold molar excess of TC10 (magenta), TC10KR/GS (black), or TC10ΔC (orange). ^***^P < 0.001. **(C)** F1_*DA*_ binding affinity plot of TC10KR/GS (black) and TC10ΔC (orange). Affinities were determined by first converting ⟨τ⟩ into the fractional saturation using Equation 4 and the data were further fitted with Equation 5. TC10KR/GS and TC10ΔC binding affinity for F1_*DA*_ were measured as 19 ± 2 μM and 13 ± 1 μM, respectively. Data from three individual biological replicates (*n* = 3) are presented as mean values ± SD. **(D)** High-FRET state and low-FRET species composition plotted against increasing concentrations of TC10KR/GS (black) and TC10ΔC (orange) obtained after analyzing the time-resolved fluorescence intensities with the Gaussian distribution model (Equations 9–11). In both cases the high-FRET state (R_1_, open square) gradually decreases, while the low-FRET (R_2_, filled square) state increases with increasing concentrations of the TC10 variants.

Therefore, we aimed to inspect the role of the basic amino acid stretch of TC10 in CB recognition and binding. To this end, we purified a TC10KR/GS variant (Supplementary Figure 1C) in which basic residues were replaced with glycine and serine as described before (Kilisch et al., 2020; Figure 3A). Additionally, we also constructed a C-terminal deletion variant of TC10 (TC10ΔC) in which residues 186-205 containing the positively charged residues were removed (Figure 3A; Supplementary Figure 1C).

For initial interaction studies, we incubated F1_*D0*_ and F1_*DA*_ with a 100-fold molar excess concentration (100 μM) of both TC10 variants and measured the change in ⟨τ⟩. Both TC10KR/GS and TC10ΔC led to a significant increase (Figure 3B and Table 1) in ⟨τ⟩ of F1_*DA*_, however, no ⟨τ⟩ change was detected in F1_*D0*_. The observed ⟨τ⟩ change for F1_*DA*_ in case of TC10KR/GS (2.89 ± 0.03 ns) and TC10ΔC (2.83 ± 0.02 ns) was comparable to the wild-type TC10 (2.87 ± 0.01 ns) (Figure 3B; Supplementary Figure 2B). The highly similar change in ⟨τ⟩ elicited by binding of TC10 C-terminal variants indicated that elimination of the phosphoinositide binding site in case of TC10KR/GS, or even the complete removal of the basic residues in TC10ΔC does not affect CB recognition by TC10.

### Deletion of TC10 C-terminal stretch enhances its affinity for collybistin

Since TC10 and its C-terminal variants induced a similar ⟨τ⟩ change in the F1_*DA*_ molecules, we next investigated whether the TC10 variants possess similar affinities for CB. To examine the binding strength of TC10 variants, we titrated F1_*DA*_ (Supplementary Figures 2C,D) separately with increasing concentrations of TC10KR/GS and TC10ΔC and quantified the results. TC10KR/GS and TC10ΔC displayed comparable binding affinities (Figure 3C) characterized by K_*d*_-values of 19 ± 2 μM and 13 ± 1 μM, respectively. Hence, the observed affinity values for the TC10 variants were roughly two-fold reduced compared to wild-type TC10 (Figure 2C) with a binding constant of 37 ± 4 μM.

To better understand the conformational changes induced in F1_*DA*_ by TC10KR/GS and TC10ΔC, we performed distance distribution fittings for both constructs as described for wild-type TC10 (Figure 2D; Supplementary Figure 3A). Both, TC10KR/GS and TC10ΔC were found to be potent (Figure 3D) in turning the high FRET F1_*DA*_ molecules into a low FRET population as seen for the wild-type TC10 (Figure 2D). The inter-fluorophore distances for the high FRET (R_1_) and low FRET (R_2_) molecules remained relatively unchanged for TC10KR/GS (R_1_ = 25.3 ± 1.9Å and R_2_ = 45.1 ± 4Å) (Supplementary Figure 3B) and TC10ΔC (R_1_ = 27.1 ± 1.1Å and R_2_ = 48.1 ± 0.6Å) (Supplementary Figure 3C) and were found to be highly similar to the TC10 wild-type (R_1_ = 26.8 ± 2.6Å and R_2_ = 47.5 ± 4.3Å) (Table 1). Higher concentrations of TC10KR/GS and TC10ΔC variants also led to a stronger population of the x_*NoFRET*_ state (Supplementary Figures 4B,C). Thus, the comparative changes in ⟨τ⟩ and the related distance distribution results for the C-terminal TC10 variants indicate a similar conformational modulation in CB, as the one elicited by the TC10 wild-type.

### Cdc42 and TC10 efficiently interact with active state mutant sensors of collybistin

Full-length CB is stabilized by intramolecular interactions between the SH3 domain and the tandem DH-PH domains (Soykan et al., 2014). The equilibrium between the inactive and active conformations in full-length CB is known to be modulated by the amino acid residues W24 and R70 in the SH3 domain, and E262 in the DH domain (Soykan et al., 2014). Earlier studies (Soykan et al., 2014) demonstrated that alanine substitutions of W24 (W24A) and E262 (E262A) weaken the intramolecular interactions and stabilize the open state of CB. Therefore, we employed open state mutant sensors (Imam et al., 2022) to investigate the interactions of TC10 and Cdc42 with CB in the open conformation.

**FIGURE 4:**
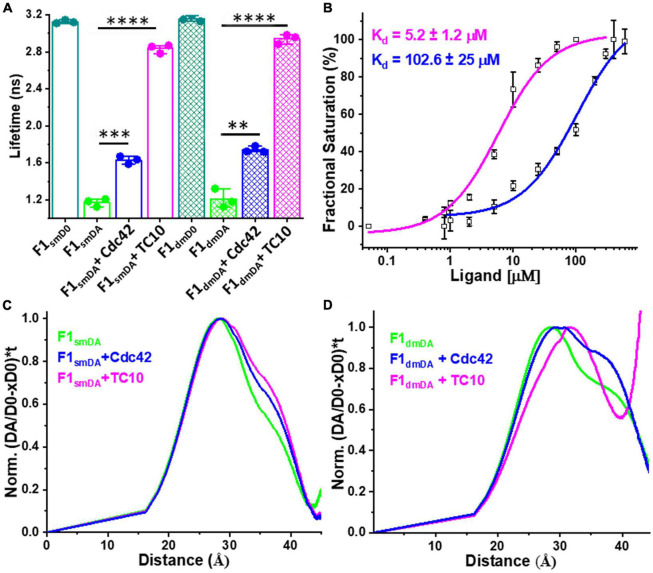
Interactions of open state mutant sensors with Cdc42 and TC10. **(A)** Bar plot depicting the species-weighted CFP ⟨τ⟩ of the CB wild-type, open state single mutant (sm) and double mutant (dm) FRET sensors (F1_*smD0*_ and F1_*dmD0*_), their FlAsH-labeled counterparts (F1_*smDA*_ and F1_*dmDA*_) and the FlAsH labeled sensors in the presence of Cdc42 or TC10. ^**^P < 0.01, ^***^P < 0.001, ^*⁣*⁣**^P < 0.0001. **(B)** F1_*dmDA*_ binding affinity (K_*d*_) plots for TC10 (magenta) and Cdc42 (blue). TC10 and Cdc42 binding affinity for the F1_*dmDA*_ were measured as 5.2 ± 1.2 μM and 102 ± 25 μM, respectively. Data from three individual biological replicates (*n* = 3) are presented as mean values ± SD. **(C,D)** Model-free distance distribution fits for the inter-fluorophore distance corresponding to the time-resolved CFP fluorescence intensities (Equations 12, 13). Normalized distance distribution curves shown for F1_*smDA*_ **(C)** and F1_*dmDA*_ **(D)** in the absence (green) and presence of TC10 (magenta) and Cdc42 (blue).

We measured the average fluorescence lifetime ⟨τ⟩ for single (F1_*smDA*_) and double mutant (F1_*dmDA*_) CB FRET sensors containing either the single W24A or double W24A/E262A amino acid replacements (Imam et al., 2022) by incubating the sensors and F1_*smD0*_ and F1_*dmD0*_ as controls with a 100-fold molar excess (100 μM) of Cdc42 and TC10. For the control measurements with F1_*smD0*_ and F1_*dmD0*_ no change in ⟨τ⟩ was observed (Supplementary Table 1). In contrast to the wild-type sensor F1_*DA*_ with a ⟨τ⟩ of 2.53 ± 0.03 ns, Cdc42 interaction with F1_*smDA*_ and F1_*dmDA*_ resulted in a significant increase in their ⟨τ⟩ to 1.63 ± 0.03 ns and 1.7 ± 0.02 ns, respectively (Figure 4A and Table 1; Supplementary Table 1). Compared to Cdc42, the interaction of TC10 with F1_*smDA*_ and F1_*dmDA*_ led to an even stronger ⟨τ⟩ increase with 2.83 ± 0.04 ns and 2.9 ± 0.04 ns, respectively (Figure 4A; Supplementary Figure 5A and Supplementary Table 1).

Since the ⟨τ⟩ change inflicted by TC10 and Cdc42 were quite significant, we next investigated their binding affinity for the open state mutant sensor and titrated F1_*dmDA*_ with increasing concentrations of TC10 and Cdc42. In both cases, rising concentrations led to a concomitant increase in ⟨τ⟩ of F1_*dmDA*_ followed by saturation (Supplementary Figures 5B,C). Interestingly, compared to the wild-type sensor (F1_*DA*_) the double mutant sensor (F1_*dmDA*_) exhibited an enhanced binding affinity for TC10 with a K_*d*_ of 5.2 ± 1.2 μM (Figure 4B) vs. a K_*d*_ of 37 ± 4 μM for F1_*DA*_ (Figure 2C). Although the titration with Cdc42 also resulted in a gradual ⟨τ⟩ increase in F1_*dmDA*_, the overall change was considerably lower than for TC10 (Supplementary Figure 5B and Supplementary Table 1) resulting in a low affinity interaction with K_*d*_ of 102.6 ± 2.5 μM for the Cdc42-CB complex (Figure 4B).

### Active state sensors display differential responses upon GTPase binding

In case of the F1_*smDA*_ and F1_*dmDA*_ sensors a rapid exponential decay of the fluorescence intensities in both sensors made the fitting with the Gaussian distance distribution model cumbersome. Thus, we relied on a model-free approach (Peulen et al., 2017) to visualize the distance distribution underlying the time-resolved fluorescence intensities of both sensors (Figures 4C,D; Supplementary Figures 7, 8). For comparison, F1_*DA*_ alone and the F1_*DA*_ complexes with TC10 and Cdc42 were analyzed in the same fashion (Supplementary Figures 6A–D). Consistent with previous results (Imam et al., 2022), model-free distance distribution analyses of F1_*smDA*_ and F1_*dmDA*_ (Figures 4C,D) yielded a main peak at around 28Å (high-FRET state) along with a small shoulder at 37Å, which was less prominent in F1_*smDA.*_ These distances depict the high and low FRET states, respectively, for the open state sensors (Figures 4C,D).

Cdc42 and TC10, upon interaction with F1_*smDA*_, resulted in an increase in the shoulder fraction at 37Å (Figure 4C). Compared to Cdc42, TC10 induced a larger increase in the shoulder (Figure 4C), thus indicating that TC10 binding leads to a more efficient increase in the inter-fluorophore distance in F1_*smDA*_. Next, we analyzed the F1_*dmDA*_ distance distribution change upon ligand interaction. Cdc42 binding to F1_*dmDA*_ resulted in a strong increase in the shoulder, located in this case at approximately 37Å (Figure 4D). The interaction of TC10 with F1_*dmDA*_ led to a rightward shift of the main peak in F1_*dmDA*_ from 28 to 32Å (Figure 4D), while the shoulder at 37Å observed in both F1_*dmDA*_ and the F1_*dmDA*_-Cdc42 complex disappeared. This was coupled to a concomitant increase in the X_*NoFRET*_ fraction as observed in the inter-fluorophore distance increase beyond 40Å (Figure 4D). The observed inter-fluorophore distance change in F1_*smDA*_ and F1_*dmDA*_ suggested that the individual sensors induce distinct conformational states after TC10 and Cdc42 binding.

### Cdc42 and TC10 induce variable responses in additional fluorescence resonance energy transfer sensors

**FIGURE 5:**
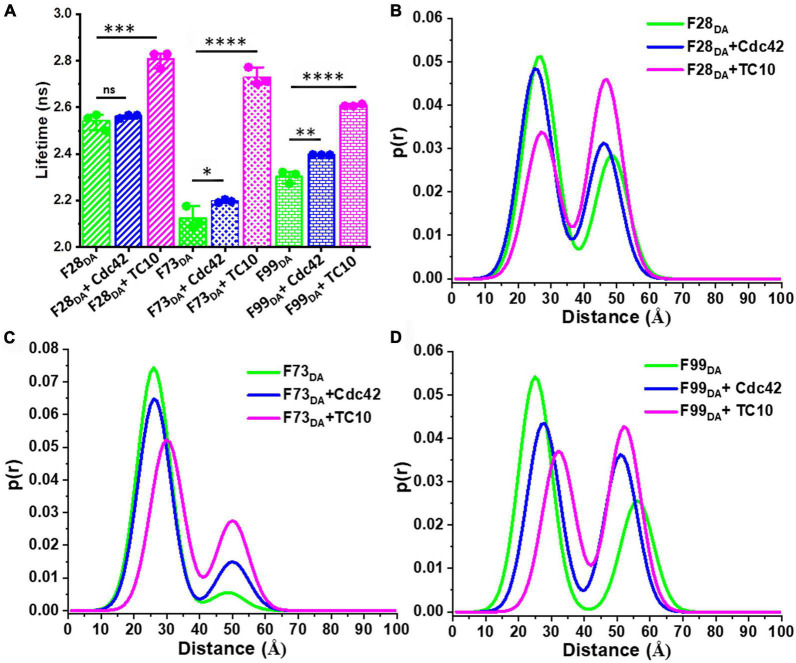
TC10 and Cdc42 induce varied responses in additional FRET sensors. **(A)** Bar graph showing the species-weighted average fluorescence-lifetime of F28_*DA*_, F73_*DA*_, and F99_*DA*_ alone (green) and in the presence of a 100-fold molar excess of Cdc42 (blue) or TC10 (magenta). *P < 0.05, ^**^P < 0.01, ^***^P < 0.001, ^*⁣*⁣**^P < 0.0001; ns, statistically not significant. **(B–D)** Plots showing the distance distribution obtained from the two Gaussian distributed distance fit model for **(B)** F28_*DA*_, **(C)** F73_*DA*_, and **(D)** F99_*DA*_ in the absence (green) and presence of Cdc42 (blue) and TC10 (magenta).

Collybistin opening disrupts the inter-domain interactions between the SH3-domain and the tandem DH-PH domain leading to dislocation of the SH3 domain (Soykan et al., 2014). We probed the SH3-domain orientation with respect to the remainder of CB following activation by both GTPases. We employed a previously described additional set of SH3-domain responsive CB FRET sensors (Imam et al., 2022) incorporating the FlAsH moiety after amino-acid residue 28 (F28_*DA*_), 73 (F73_*DA*_), and 99 (F99_*DA*_) (Supplementary Figure 1A) and measured ⟨τ⟩ in the absence and presence of a 100-fold molar excess of TC10 and Cdc42 (Figure 5A). For F28_*DA*_ no ⟨τ⟩ change was observed (Figure 5A; Supplementary Table 2) upon interaction with Cdc42 (⟨τ⟩ = 2.56 ± 0.01 ns) whereas TC10 caused a substantial increase (⟨τ⟩ = 2.8 ± 0.03 ns). F73_*DA*_ showed a minute ⟨τ⟩ increase in the presence of Cdc42 (⟨τ⟩ = 2.19 ± 0.005 ns) and a considerable increase with TC10 (⟨τ⟩ = 2.73 ± 0.03 ns) (Figure 5A; Supplementary Table 2). Similar to F73_*DA*_ a minor increase in (⟨τ⟩ = 2.39 ± 0.01 ns) was observed with Cdc42 for F99_*DA*_ (Figure 5A; Supplementary Table 2), while TC10 led to a slightly smaller increase (⟨τ⟩ = 2.6 ± 0.004 ns) in F99_*DA*_ compared to the other sensors (Figure 5A; Supplementary Table 2).

We also carried out distance distribution studies for all sensors in the absence and presence of a 100-fold molar excess of Cdc42 and TC10. Consistent with our previous study (Imam et al., 2022), all sensors displayed comparable inter-fluorophore distances in the absence of ligands. Cdc42 could not change the equilibrium between the high-FRET (x_1_) and low-FRET states (x_2_) in the F1_*DA*_ and F28_*DA*_ sensors (Figure 5B; Supplementary Table 2). However, Cdc42 addition led to significant changes in the x_1_ and x_2_ species in F73_*DA*_ and F99_*DA*_ (Figures 5C,D; Supplementary Table 2), hence shifting the equilibrium toward the low-FRET state. In contrast to Cdc42, TC10 addition led to a strong shift in the equilibrium from the high-FRET to the low-FRET state in all (F1_*DA*_, F28_*DA*_, F73_*DA*_, and F99_*DA*_) sensors (Figures 5B–D; Supplementary Table 2). The overall results evidently suggest that both GTPases occupy different binding sites (Xiang et al., 2006; Mayer et al., 2013) relative to the respective sensor and hence induce variable responses in the different sensors.

### Cdc42 and TC10 display different electrostatic potentials

**FIGURE 6:**
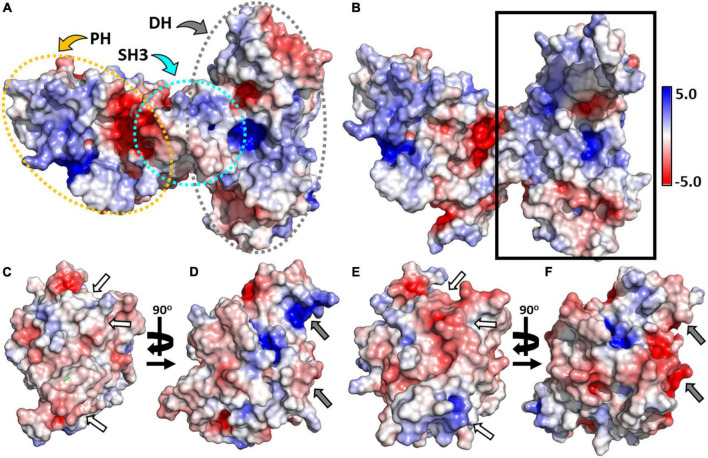
Electrostatic potentials of CB, Cdc42, and TC10. **(A)** Surface representations showing the electrostatic potentials of full-length CB (PDB entry 4mt6). The SH3, DH, and PH domains are outlined by dotted ellipsoids shown in cyan, gray, and yellow, respectively. **(B)** Electrostatic potential of CBSH3^–^ (PDB entry 2dfk) viewed into the interface region (solid rectangle) of the CBSH3^–^-Cdc42 complex with Cdc42 omitted from the calculation. **(C,E)** Electrostatic potential of Cdc42 showing the interface region of the Cdc42-CBSH3^–^ complex after rotation by 180° around the vertical axis **(C)** and hypothetical CB-TC10 interface region following superimposition of TC10 on Cdc42 **(E)**. Regions possessing substantial charge differences between Cdc42 **(C)** and TC10 **(E)** are emphasized by white arrows. **(D,F)** Surface charge potential of Cdc42 **(D)** and TC10 **(F)** when rotated by 90° around the vertical axis. Sections having substantial charge differences between Cdc42 **(D)** and TC10 **(F)** are highlighted by gray arrows. All electrostatic potentials are represented by isosurfaces contoured at –5.0 k_*b*_T/e_*c*_ (red) or 5.0 k_*b*_T/e_*c*_ (blue), respectively.

To better understand the molecular basis of differential recognition of both GTPases by CB, we calculated the electrostatic potential of the two proteins (Hemsath et al., 2005; Xiang et al., 2006; Soykan et al., 2014) using APBS (Jurrus et al., 2018) at an ionic strength of 150 mM. Full-length CB was found to contain small patches of positive, neutral, and negative residues, uniformly distributed over the surface of the SH3 and DH domains (Figure 6A). In contrast, the PH domain possesses a positively charged area in close proximity to the SH3-PH domain interface (Figure 6A). Removing Cdc42 from the complex with the SH3-domain truncated CB variant (Figure 6B) illustrated that the top interface region located in the DH domain largely consists of positively charged residues, whereas the bottom section contained a small patch of acidic residues.

Analysis of the Cdc42 interface region in the Cdc42-CBSH3^–^ complex (Figure 6C) revealed no prominent electronegative or electropositive features, thus indicating that complex formation is driven by hydrophobic interactions and hydrogen bonds as outlined above (Figures 1D,F). In contrast, the corresponding surface of TC10 (Figure 6E) contains strong negative patches at its center and a smaller patch of positively charged electrostatic potential at the bottom. Surprisingly, these patches are complementary to those observed in CB where Cdc42 interacts. Hence, the inability of TC10 to interact with CB in an analogous manner as Cdc42 must arise from the amino acid replacements discussed earlier (Figures 1D–G), which abrogate the hydrophobic contacts and H-bonds present in the CB-Cdc42 complex. Rotation of both GTPases by 90° highlighted additional differences; TC10 featured a significant electronegative patch, in contrast to Cdc42 with an electropositive patch at the same region (right edge in Figures 6D,F). This region in TC10 would be ideally suited to interact with the positively charged PH domain, in line with its known binding preference (Mayer et al., 2013). At the same time, Cdc42 cannot interact with the PH domain in the same manner since it is oppositely charged in this region.

## Discussion

Activation of Ras-related GTPases and their isoforms induces a plethora of cellular processes, including reorganizations of the actin cytoskeleton governing the cell cycle and cellular motility (Hall, 1998; Hodge and Ridley, 2016; Mosaddeghzadeh and Ahmadian, 2021). In humans, based on sequence similarity, 20 canonical members of the Rho family have been identified to date (Wittinghofer and Vetter, 2011). The GTPases belonging to the Cdc42 subfamily, TC10 and Cdc42, share common cellular functions (Murphy et al., 2001), however, TC10 expression is limited to specific hippocampal regions (Tanabe et al., 2000) where the most prominent reduction in gephyrin is observed in CB knock-out mice (Papadopoulos et al., 2007), thus suggesting a potential role in GABA_*A*_ receptor clustering (Mayer et al., 2013).

Previous cell-based and biochemical studies documented that TC10 binding to CB triggers synaptic gephyrin clustering and enhances GABAergic neurotransmission (Mayer et al., 2013; Kilisch et al., 2020). Moreover, prior work demonstrated that CB interaction with the intracellular domain of NL2 or Cdc42 leads to an open structure of CB, which favors its interaction with phosphoinositides located in the postsynaptic membrane (Poulopoulos et al., 2009; Soykan et al., 2014; Schäfer et al., 2020). Our study with the wild-type mimicking CB FRET sensor (F1_*DA*_) (Supplementary Figure 1A) upon interaction with TC10 resulted in a significant increase in ⟨τ⟩ (Figures 2A,B), indicating a TC10-mediated CB opening. In contrast, the inability of Cdc42 to induce any ⟨τ⟩ change in F1_*DA*_ reflects the preferential binding of CB to TC10. Furthermore, we could determine the binding strength of the CB-TC10 complex with a K_*d*_ of 37 ± 4 μM, which so far had not been determined (Figure 3C). The interaction with Cdc42 is considerably weaker, which precluded an experimental determination of the binding strength by our approach.

The C-terminal extension of TC10 harbors several basic residues, which have been shown to play an important role in CB-dependent gephyrin micro-clustering (Kilisch et al., 2020). A TC10 variant in which several lysine and arginine C-terminal residues were replaced with glycine and serine (TC10KR/GS) failed to stimulate gephyrin clustering and abrogated phosphoinositide binding (Kilisch et al., 2020). Our studies showed that the TC10KR/GS variant bound more tightly (K_*d*_ = 19 ± 2 μM) as did the TC10ΔC variant in which the C-terminal residues were removed (K_*d*_ = 13 ± 1 μM), compared to the TC10 wild-type (K_*d*_ = 37 ± 4 μM) (Figures 2C, 3C). This demonstrated that, while the C-terminal residues are crucial for phosphoinositide-binding (Kilisch et al., 2020), they do not contribute to TC10-CB complex formation *in vitro*. In fact, our electrostatic analysis suggested that the interaction between TC10 and the PH domain of CB is driven by electrostatic interactions with the PH domain being positively charged and TC10 being negatively charged. The presence of additional positive charges at the TC10 C-terminus could hence weaken this electrostatic complementarity.

**FIGURE 7:**
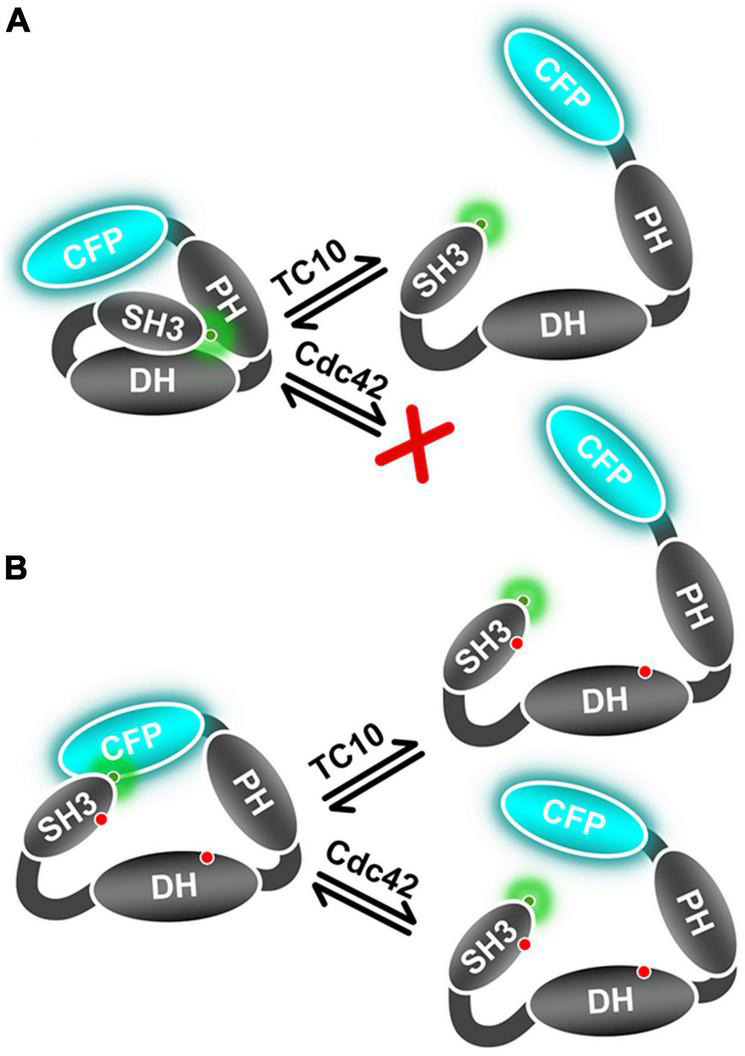
Schematic representation of TC10 and Cdc42 mediated CB conformational activation. **(A)** This panel represents the wild-type mimicking CB FRET sensor (F1_*DA*_) in the auto-inhibited form and its conformational state after interaction with TC10 or the inability of Cdc42 to interact with this sensor. **(B)** Cartoon depicting the active state CB FRET sensor (F1_*dmDA*_) conformational change after TC10 and Cdc42 binding. TC10 binding induces a strong change in ⟨τ⟩ and hence a large inter-fluorophore movement, whereas it is relatively small for Cdc42. Red dots on the SH3 and DH domain represent the incorporated amino-acid replacements in the F1_*dmDA*_ construct.

The Cdc42 interaction with the open state mutant sensors F1_*smDA*_ and F1_*dmDA*_, as reflected in the ⟨τ⟩ increase (Figure 4A), suggested that Cdc42 only binds to the open-state CB. Quantification of the F1_*dmDA*_ data revealed a rather low affinity characterized by a K_*d*_-value of 102 ± 25 μM (Figure 4B), in contrast to the Cdc42-F1_*DA*_ interaction where no binding could be detected (Figures 2A,B). This finding further corroborated previous biochemical data (Xiang et al., 2006), which revealed that full-length CB showed a significantly reduced GEF activity compared to the CB2-SH3^–^ variant. A similar trend was also observed for TC10 where binding to both F1_*smDA*_ and F1_*dmDA*_ sensors led to a strong ⟨τ⟩ increase (Figure 4A), which resulted in an increase in binding affinity (K_*d*_ = 5.2 ± 1.2 μM) to the F1_*dmDA*_ sensor (Figure 4B). The preferential binding of Cdc42 to F1_*dmDA*_ and the enhanced binding of TC10 to this sensor presumably reflects an increased accessibility of the respective binding site. While this can be straightforwardly understood in the case of Cdc42 where the SH3 domain in the closed state of CB (Soykan et al., 2014) partially overlaps with Cdc42 (Figure 6A), it cannot be easily rationalized for the CB-TC10 interaction in the absence of structural data. One possible explanation would be that the SH3 domain in the closed conformation slightly overlaps with the TC10 binding site in the PH domain (Mayer et al., 2013; Kilisch et al., 2020). Based on our time-resolved fluorescence-based FRET data, we propose a simplified model (Figure 7) for GTPase-mediated conformational activation of CB.

In summary, this study provides clear evidence of a TC10-induced CB conformational switch from its auto-inhibited or closed state to an open/active state. As described earlier (Soykan et al., 2014), the open conformation is critical for the ability of CB to promote the formation of inhibitory postsynaptic structures. Despite the fact that Cdc42 is a closely related GTPase, it fails to induce this conformational change in full-length CB, which, on the molecular level, correlates with its entirely different mode of interaction with CB. Contrary to the ubiquitous expression of Cdc42, the limited expression of TC10 in the hippocampus was reported to be essential for CB-dependent gephyrin clustering (Mayer et al., 2013; Kilisch et al., 2020). Our data hence suggest that the TC10-induced stabilization of CB in the open state is critical for gephyrin clustering. Interestingly, both GTPases have also been reported to interact with another Dbl family Rho GEF, ARHGEF7 (also called βPix) *via* its catalytic DH domain (Feng et al., 2002; López Tobón et al., 2018). Intriguingly, βPix-deficient neurons lack the ability of axon formation in culture and in the developing cortex. Nevertheless, the loss can be rescued by the expression of TC10, but not Cdc42 (López Tobón et al., 2018). Since there are no reports regarding GTPase-mediated βPix conformational activation, it would be interesting to investigate as to how TC10 and Cdc42 interact with βPix and whether they possibly induce similar conformational changes as observed for CB in this study.

## Data availability statement

The raw data supporting the conclusions of this article will be made available by the authors, without undue reservation.

## Author contributions

NI generated the TC10 constructs, purified the proteins, carried out *in vitro* experiments, and performed the time-resolved fluorescence measurements. NI and SC analyzed the results. NI and HS prepared the manuscript. HS and KH supervised the project. All authors contributed to the article and approved the submitted version.

## Footnotes

1
https://www.mpc.hhu.de/software/3-software-package-for-mfd-fcs-and-mfis
