# Supplementary material for: Differential modulation of collybistin conformational dynamics by the closely related GTPases Cdc42 and TC10
Source: Front Synaptic Neurosci. 2022 Aug 4;14:959875. doi: 10.3389/fnsyn.2022.959875 (PMC9386560; doi:10.3389/fnsyn.2022.959875)
Supplement: Supplementary file 1 [file Data_Sheet_1.pdf]

Supplementary Material

Differential modulation of collybistin conformational dynamics

by the closely related GTPases Cdc42 and TC10

Nasir Imam,[1] Susobhan Choudhury,[2] Katrin G. Heinze,[2]* Hermann Schindelin[1]*

[1] Institute of Structural Biology, Rudolf Virchow Center for Integrative and Translational Bioimaging, University of Würzburg, Josef-Schneider Str. 2, 97080 Würzburg, Germany.

[2] Molecular Microscopy, Rudolf Virchow Center for Integrative and Translational Bioimaging, University of Würzburg, Josef-Schneider Str. 2, 97080 Würzburg, Germany.

* Correspondence: hermann.schindelin@virchow.uni-wuerzburg.de
or
katrin.heinze@virchow.uni-wuerzburg.de

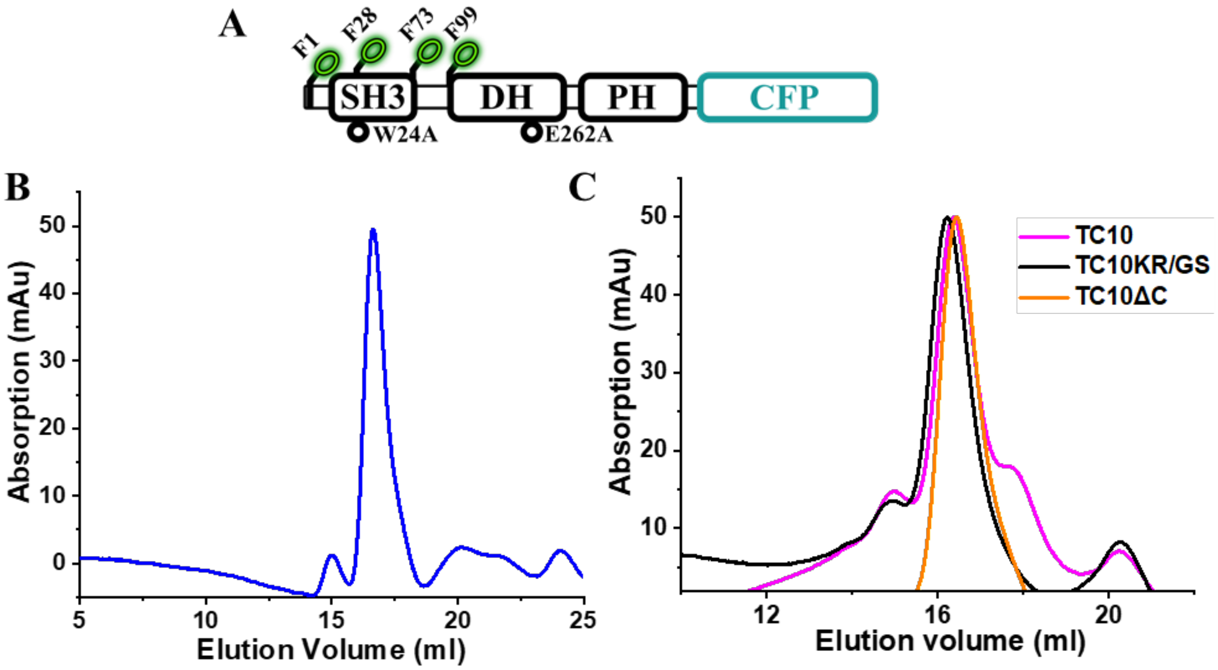

Supplementary Figure 1. CB FRET sensor construction and GTPases purification. (A) Set of CB FRET sensors used in this study. The inserted tetra-cysteine motifs (tCM) used for labeling with the fluorescein arsenical hairpin binder-ethanedithiol (FlAsH-EDT_2) are represented as green loops and the C-terminally attached CFP is shown in teal. Individual sensors comprise single tCM inserted after residue 1 (F1), 28 (F28), 73 (F73) and 99 (F99). Black dots in the SH3 and DH domain represent the amino acid replacement sites for the single and double mutant FRET sensor ($F1_{smDA}$ and $F1_{dmDA}$). **(B)** Size exclusion chromatography elution profile of Cdc42 (blue). **(C)** Elution profile of TC10 (magenta) and its C-terminal variants TC10KR/GS (black) and TC10ΔC(orange).

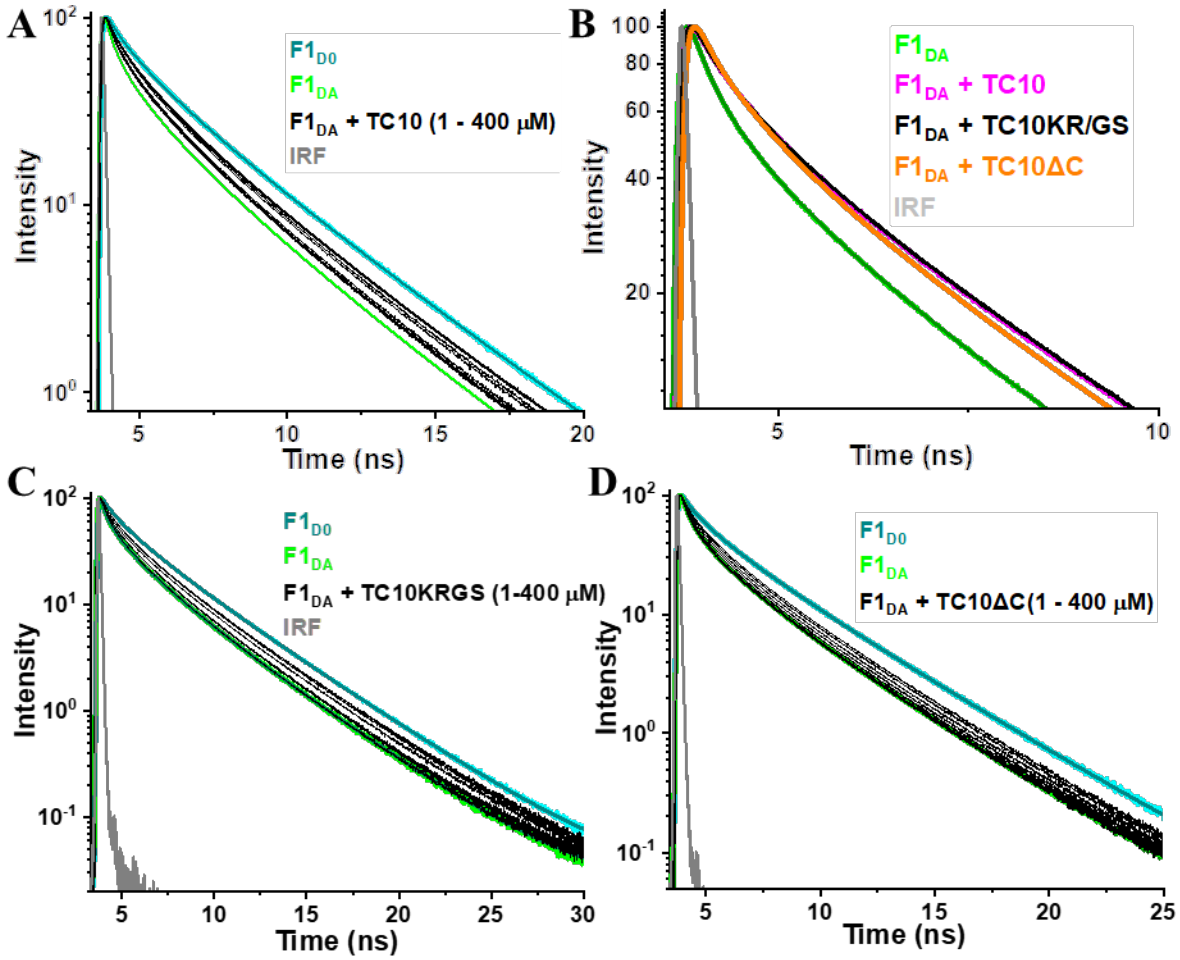

Supplementary Figure 2. Titrations of TC10 and its C-terminal variants with F1$_{DA}$. (A) Fluorescence lifetimes of CFP in F1$_{D0}$ (teal), F1$_{DA}$ (green) and F1$_{DA}$-TC10 complexes with increasing TC10 concentrations (black). **(B)** Time-resolved fluorescence intensities of CFP of FlAsH-labeled CB FRET sensor (F1$_{DA}$) in the absence (green) and presence of TC10 (magenta), TC10KR/GS (black) and TC10ΔC (orange). (**C-D)** Fluorescence lifetimes of CFP in F1$_{D0}$ (teal), F1$_{DA}$ (green) and F1$_{DA}$ with increasing concentrations of TC10KR/GS (C; black) and TC10ΔC (D; black). Data in A-D are scaled to a maximum of 10^2 for easier comparison.

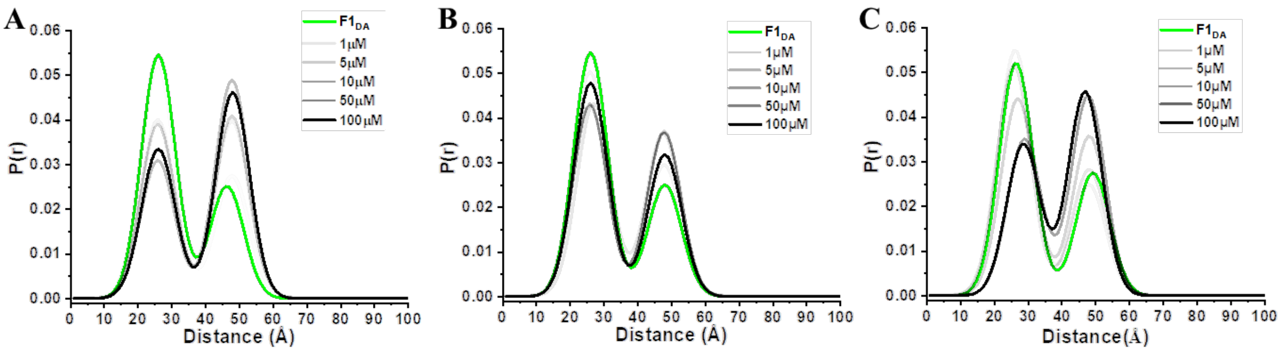

Supplementary Figure 3. Gaussian distance distribution analysis of F1$_{DA}$ with TC10 and its variants. (A-C) Distance distribution plots of F1$_{DA}$ (green) upon interaction with increasing concentrations of TC10 (A), TC10KR/GS (B) and TC10ΔC (C). Increasing concentrations of TC10 and its C-terminal variants (TC10KR/GS and TC10ΔC) lead to a gradual decrease in the high-FRET state and concomitant increase in the low-FRET state.

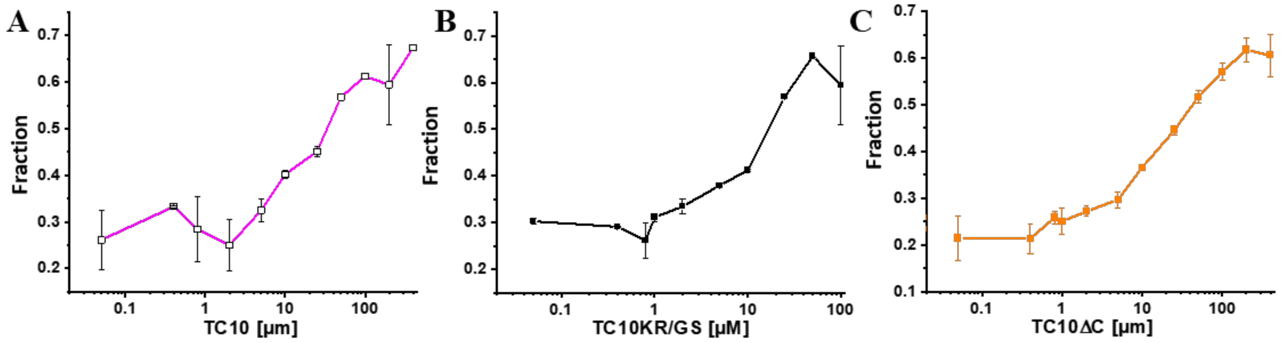

Supplementary Figure 4. **No-FRET fractions after fitting with the Gaussian distribution model.** **(A-C)** Increase in the No-FRET fraction (x_{noFRET}) of $F1_{DA}$ with increasing molar concentrations of TC10 (A), TC10KR/GS (B) and TC10ΔC (C). These data indicate the existence of an additional state exhibiting an inter-fluorophore distance >49 Å.

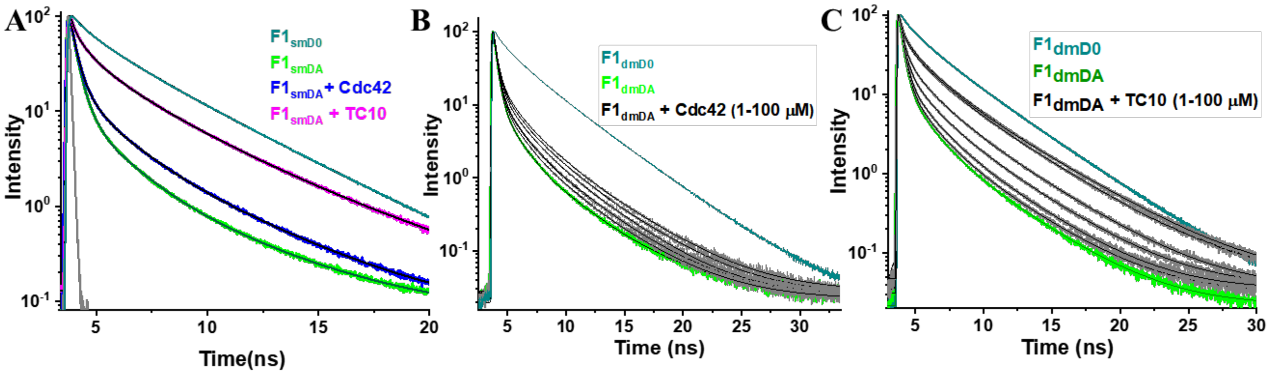

Supplementary Figure 5. Interactions of the open state mutant sensors with TC10 or Cdc42. (A) CFP fluorescence intensity in single mutant sensor ($F1_{smD0}$; teal), its FlAsH labeled counterpart ($F1_{smDA}$) alone (green) and in the presence of either Cdc42 (blue) or TC10 (magenta). The instrument response function (IRF) is shown in grey. **(B)** Fluorescence lifetime decay of CFP in the double mutant FRET sensor with $F1_{dmD0}$ (teal), $F1_{dmDA}$ alone (green) and $F1_{dmDA}$-Cdc42 complexes with increasing concentrations of Cdc42 (black). **(C)** $F1_{dmDA}$ alone (green) and $F1_{dmDA}$-TC10 complexes with increasing concentrations of TC10 (black). Data in A-C are scaled to a maximum of 10^2 for easy comparison.

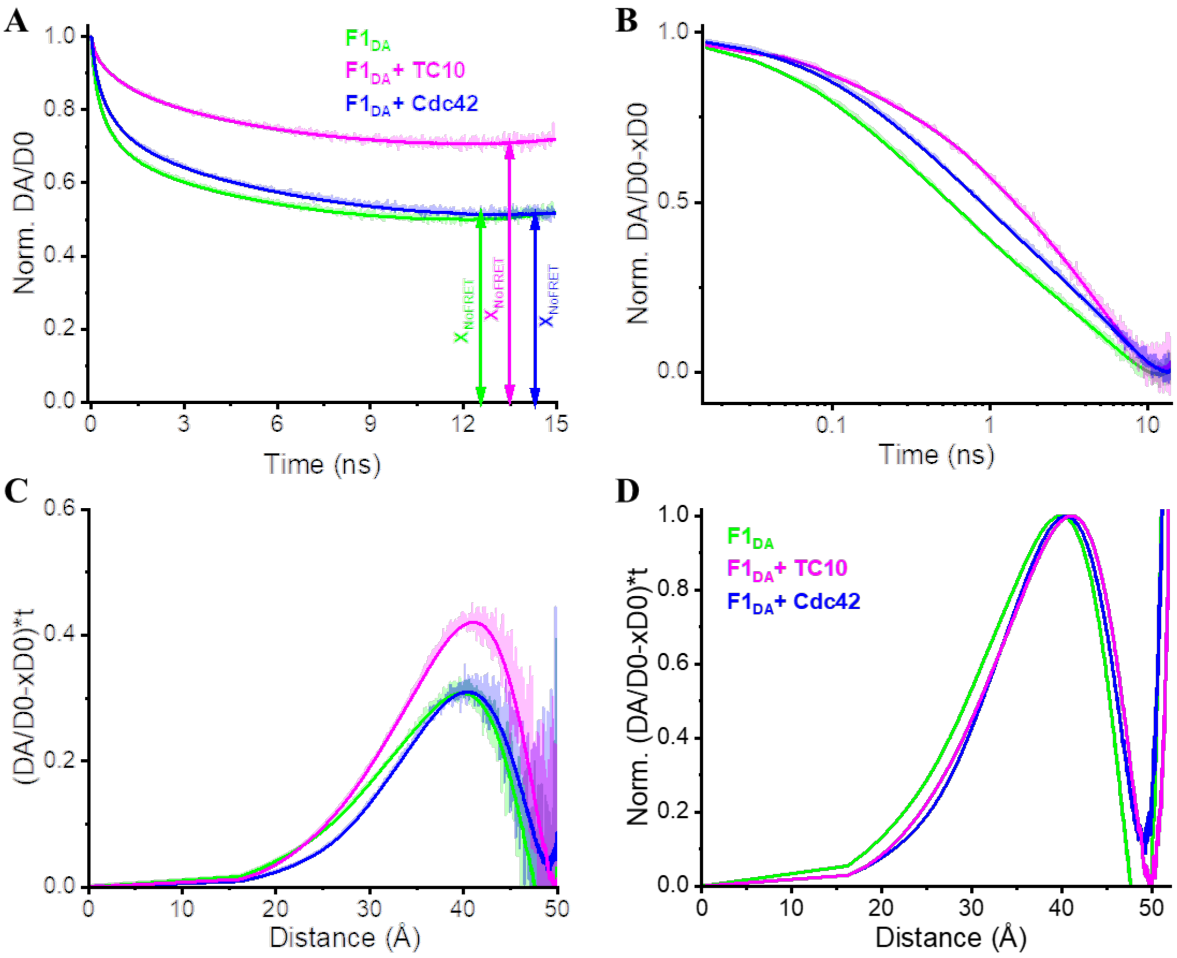

Supplementary Figure 6. Model-free distance distribution analysis of F1$_{DA}$. (A) Time-resolved fluorescence intensities $I_{F1DA}(t)$ of the wild type mimicking FlAsH labeled sensors alone (green) and in the presence of a 100-fold molar excess of TC10 (magenta) and Cdc42 (blue) divided by $I_{F1D0}(t)$, the corresponding intensity without FlAsH labeling. The off-set values of each curve represent the x_{NoFRET} fraction. **(B)** The offset in (A) is subtracted and time is displayed on a logarithmic scale. **(C)** The time-axis is converted to a distance axis (eqs. 12-13; main text). **(D)** The probability density distribution of the underlying distance distribution is normalized to 1 for easier comparison.

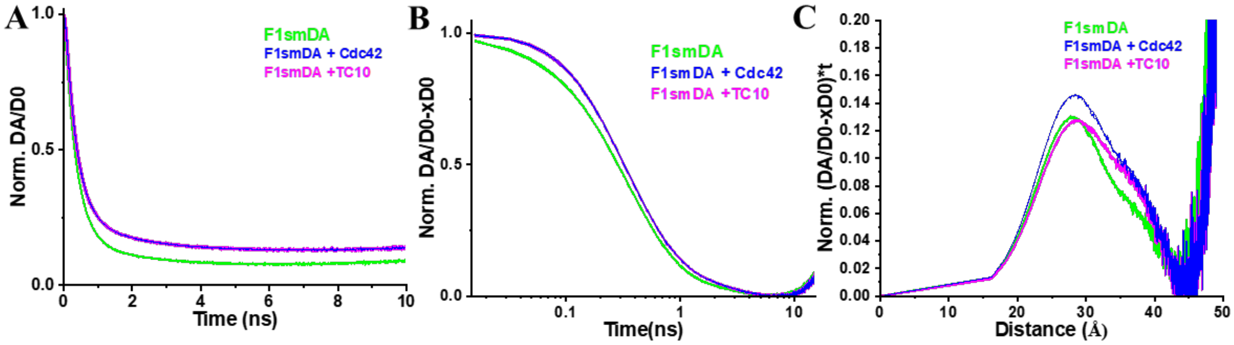

Supplementary Figure 7. Model-free distance distribution of time-resolved fluorescence intensities of the open state single mutant sensor (F1$_{\mathbf{smDA}}$). (A) Time-resolved fluorescence intensities of the FlAsH-labeled single mutant sensor, $I_{F1smDA}(t)$, alone (green) and in the presence of a 100-fold molar excess of TC10 (magenta) and Cdc42 (blue) divided by $I_{F1smD0}(t)$, the corresponding intensity of the singly labeled sample. The off-set values of each curve represent the x_{NoFRET} fraction. **(B)** The offset in (A) is subtracted and the time scale is logarithmic. **(C)** The time-axis is converted to the distance axis (eqs. 12-13; main text).

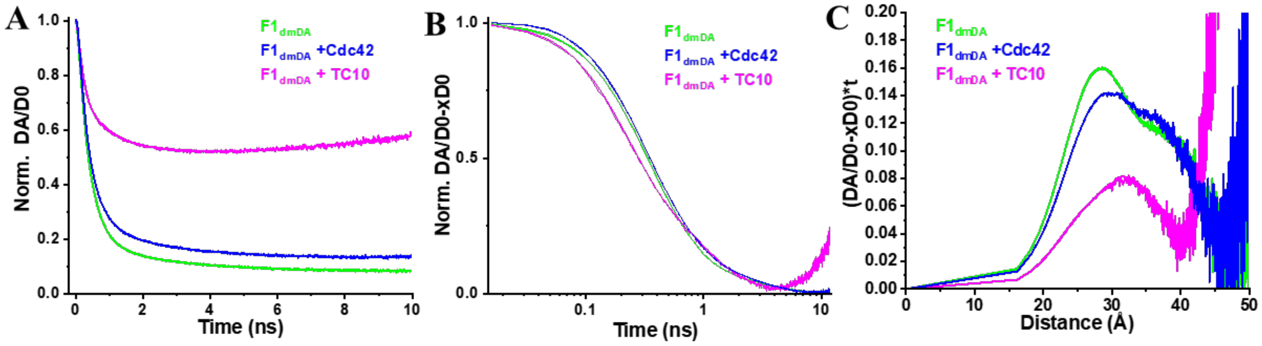

Supplementary Figure 8. Model-free distance distribution of time-resolved fluorescence intensities of the open state double mutant sensor (F1$_{\mathbf{dmDA}}$). (A) Time-resolved fluorescence intensities of the FlAsH-labeled double mutant sensor, $I_{F1dmDA}(t)$, alone and in the presence of a 100-fold molar excess of Cdc42 (blue) and TC10 (magenta) divided by $I_{F1dmD0}(t)$, the corresponding intensity of the singly labeled sample. The off-set values of the curves represent the x_{NoFRET} fraction. **(B)** The offset in (A) is subtracted and the time scale is logarithmic. **(C)** The time-axis is converted to the distance axis (eqs. 12-13; main text).

**Sample**	**⟨τ⟩ (±SD) ns**
$F1_{smD0}$	3.12 (±0.02)
$F1_{smDA}$	1.17 (±0.03)
$F1_{smDA}$ + Cdc42	1.63 (±0.03)
$F1_{smDA}$ + TC10	2.83 (±0.04)
$F1_{dmD0}$	3.15 (±0.02)
$F1_{dmDA}$	1.2 (±0.08)
$F1_{dmD0}$ + Cdc42	3.1 (±0.03)
$F1_{dmD0}$ + TC10	3.12 (±0.01)
$F1_{dmDA}$ + Cdc42	1.7 (±0.02)
$F1_{dmDA}$ + TC10	2.9 (±0.04)

Supplementary Table 1. Average fluorescence lifetime (⟨τ⟩) of open state single ($F1_{smD0}$) and double mutant ($F1_{dmD0}$) CB FRET sensors, their FlAsH labeled counterparts $F1_{smDA}$ and $F1_{dmDA}$ in the absence and presence of Cdc42, TC10 and its variants. Data from three individual biological replicates (n = 3) are presented as mean values ± SD.

Sample	⟨τ⟩ (±SD), [ns]	R_1 (±SD) [Å]	X_1 (±SD)	R_2 (±SD) [Å]	X_2 (±SD)	x_{NoFRET} (±SD)
$F1_{DA}$	2.52 (±0.02)	25.5 (±1.5)	0.45 (±0.02)	45.5 (±0.9)	0.21 (±0.02)	0.32 (±0.03)
$F1_{DA}$+ Cdc42	2.53 (±0.03)	26.2 (±1.2)	0.48 (±0.02)	42.5 (±1.9)	0.21 (±0.04)	0.29 (±0.11)
$F1_{DA}$ + TC10	2.87 (±0.01)	26.8 (±2.6)	0.20 (±0.01)	47.5 (±4.3)	0.23 (±0.09)	0.68 (±0.18)
$F28_{DA}$	2.54 (±0.03)	25.8 (±1.1)	0.47 (±0.02)	48.3 (±0.4)	0.24 (±0.02)	0.27 (±0.01)
$F28_{DA}$ + Cdc42	2.56(±0.01)	24.6 (±0.8)	0.40 (±0.01)	45 (±0.8)	0.25 (±0.01)	0.33 (±0.01)
$F28_{DA}$ + TC10	2.8 (±0.03)	27.1 (±0.1)	0.18 (±0.01)	46.7 (±0.02)	0.25 (±0.01)	0.56 (±0.01)
$F73_{DA}$	2.12 (±0.04)	24.2 (±1.1)	0.78 (±0.01)	45.6 (±0.2)	0.11 (±0.01)	0.11 (±0.06)
$F73_{DA}$ + Cdc42	2.19 (±0.01)	25.3 (±0.3)	0.63 (±0.01)	44.8 (±0.5)	0.16 (±0.01)	0.16 (±0.02)
$F73_{DA}$ + TC10	2.73 (±0.03)	22.5 (±0.3)	0.30 (±0.02)	39.6 (±1.5)	0.22 (±0.02)	0.46 (±0.01)
$F99_{DA}$	2.3 (±0.02)	24.7 (±2.1)	0.73(±0.01)	54 (±1.6)	0.12 (±0.12)	0.13 (±0.03)
$F99_{DA}$ + Cdc42	2.39 (±0.01)	25.8 (±2.3)	0.38 (±0.01)	51.7 (±1.6)	0.23 (±0.11)	0.38 (±0.07)
$F99_{DA}$ + TC10	2.6 (±0.01)	32.9 (±0.4)	0.10 (±0.03)	52.7 (±2.0)	0.14 (±0.03)	0.74 (±0.01)

Supplementary Table 2. Time-resolved FRET analysis for different CB-FRET sensors having the FlAsH moiety at positions 1, 28, 73, or 99 of CB in the presence of a 100-fold molar excess of Cdc42 or TC10. The table depicts their measured average fluorescence lifetimes (⟨τ⟩), inter-fluorophore distances (R_i) and their relative species fractions (x_i). Species fractions are normalized such that $x_1 + x_2 + x_{noFRET} = 1$. Data from three individual biological replicates (n = 3) are presented as mean values ± SD.
